# Phenotypic and Genotypic Characterization of Extended-Spectrum Beta-Lactamases Produced by *Escherichia coli* Colonizing Pregnant Women

**DOI:** 10.1155/2020/4190306

**Published:** 2020-01-23

**Authors:** Nahed Ghaddar, Elie Anastasiadis, Rawad Halimeh, Ali Ghaddar, Ghassan M. Matar, Antoine Abou Fayad, Nour Sherri, Rita Dhar, Wadha AlFouzan, Hoda Yusef, Mira El Chaar

**Affiliations:** ^1^Faculty of Science, Biological Sciences Department, Beirut Arab University, Beirut, Lebanon; ^2^Department of Obstetrics and Gynecology, Saint George Hospital, Beirut, Lebanon; ^3^Faculty of Medicine, University of Balamand, Beirut, Lebanon; ^4^Department of Biomedical Sciences, Lebanese International University, Beirut, Lebanon; ^5^Dept. of Experimental Pathology, Immunology and Microbiology, Center for Infectious Diseases Research, WHO Collaborating Center for Reference & Research on Bacterial Pathogens, Faculty of Medicine, American University of Beirut, Beirut, Lebanon; ^6^Microbiology Unit, Department of Laboratories, Farwania Hospital, Kuwait; ^7^Department of Microbiology, Health Sciences Center, Kuwait University, Kuwait; ^8^Faculty of Health Sciences, University of Balamand, Beirut, Lebanon

## Abstract

**Introduction:**

Infections caused by extended spectrum beta lactamase (ESBL) producing bacteria continue to be a challenge for choosing the appropriate therapy since they may exhibit coresistance to many other classes of antibiotics. The aim of the study was to screen pregnant women for ESBL producing bacteria in Beirut, Lebanon, to examine their phenotypic and genotypic characterization and to study the association between ESBL colonization with adverse neonatal outcomes.

**Method:**

In this cross-sectional study, vaginal samples from 308 pregnant women at 35–37 weeks of gestation were studied during a one-year period. The samples were plated on MacConkey agar and selective MacConkey agar supplemented with ceftazidime. Phenotypic confirmation of ESBL production was performed by double-disc synergy test and all isolates were screened by PCR for the resistance genes bla_SHV_, bla_TEM_, and bla_CTX-M_. Clonal relatedness of *Escherichia coli* isolates was investigated by pulsed-field gel electrophoresis.

**Results:**

In total, 59 women out of 308 (19.1%) were colonized by ESBL producing gram negative bacteria. Two babies born to mothers colonized with ESBL were diagnosed with sepsis. The susceptibility rates of isolates to other antibiotics were 39% to co-trimoxazole, 49.2% to ciprofloxacin, 91.5% to gentamicin, 18.6% to aztreonam and 35.6% to cefepime. Most of isolates were highly sensitive to meropenem and imipenem, with a susceptibility of 93.2%. PCR was performed on all *E. coli* isolates to detect the most common ESBL producing genes; bla_CTX-M _was the predominant gene (90.7%), followed by bla_TEM_ (88.4%) and finally bla_SHV_ (44.2%). PFGE analysis of 34 *E. coli* isolates revealed 22 distinct clusters showing more than 85% similarity.

**Conclusion:**

In conclusion, this study showed that Lebanon has a high prevalence of ESBL carriage in pregnant women. Further studies that include a continuous screening of pregnant women and follow up of their newborn clinical status should be conducted to foresee the risk of transmission.

## 1. Introduction

Neonatal sepsis is a blood infection that occurs during the first month after birth. Early onset neonatal sepsis (EOS) is considered the main cause of mortality and morbidity in neonates [1]. It occurs in the first three days of life and usually transmitted from mother to baby during delivery [1]. The infection may be also transmitted vertically when the amniotic membrane ruptures or prior to the onset of labor causing intra amniotic infection [1, 2]. The organisms most frequently associated with EOS are *Streptococcus agalactiae* and *Escherichia coli* [1, 2]. The latter accounts for about 24% of all EOS; 81% of cases occurring in preterm infants [1]. High rate of colonized extended spectrum beta lactamase producing *Enterobacteriaceae* in the maternal vaginal canal, in particularly *Escherichia coli* and *Klebsiella pneumonia*, have been also detected in infected newborns [3, 4]. Therefore, transmission to newborn may occur intrapartum and EOS secondary to *E. coli* may cause bacteremia with or without meningitis at the time of delivery [1].

Infections caused by ESBL producing bacteria continue to be a challenge for choosing the appropriate therapy since they may exhibit coresistance to many other classes of antibiotics [5, 6]. Sparse information exists in the literature regarding ESBL producing bacteria colonization in pregnant women. It is therefore important to know the prevalence of these microorganisms in specific geographic location and formulate an appropriate screening policy.

The aim of the study was to screen pregnant women for ESBL producing bacteria in Beirut, Lebanon and to examine their phenotypic and genotypic characterization. The study also aimed to explore the association between ESBL colonization with adverse neonatal outcomes.

## 2. Material and Methods

### 2.1. Study Population

A cross-sectional descriptive study was conducted from March 2016 to March 2017 involving 308 pregnant women at 35–37 weeks of gestation who were examined during antenatal checkup at different obstetrics and gynecology clinics in and around Beirut. Women signed a consent form to approve their participation in the study. One vaginal swab was collected by the attending physician from each patient attending the clinic for antenatal care. The samples were stored in Stuart medium (Oxoid, UK) at room temperature until transported to clinical diagnostic laboratory.

### 2.2. Data Collection

Socio-demographic data, clinical status, and gestational history of 165 (55%) patients were collected through a questionnaire by the gynecologists. The questionnaire was developed to measure women age, education, health and delivery-related variables (delivery type and delivery time, gestational diabetes, anemia, previous miscarriage, urinary tract infection, induced labor, and contact with animals as independent variables. The questionnaire also measured neonatal outcomes (neonatal weight, height and Apgar score) as dependent variables. The questionnaire was filled by the physician who examined participants. Anonymity and confidentiality were guaranteed and a written informed consent was signed by the participants. This study was approved by the institution review board of Beirut Arab University.

### 2.3. Bacterial Isolation and Identification

The clinical samples were plated on MacConkey agar and selective MacConkey agar supplemented with 1 mg/L of ceftazidime (CAZ). Plates were incubated at 37°C under aerobic conditions and examined after 24 and 48 h incubation. All isolates were identified by their culture characteristics, standard biochemical tests, and confirmed by API 20E (Biomerieux, Mary l'Etoile, France) according to the manufacturer's instructions.

### 2.4. Phenotypic Screening for ESBL

Antibiotic susceptibility testing of the collected isolates was performed by Kirby-Bauer disc diffusion method on Mueller Hinton agar following Clinical Laboratory Standard Institute (CLSI) recommendations. The antibiotic discs (Hi-Media, India) used were, gentamicin (GN, 10 *μ*g), ceftazidime (CAZ, 30 *μ*g), cefotaxime (CTX, 30 *μ*g), ciprofloxacin (CIP, 5 *μ*g), cotrimoxazole (SXT, 25 *μ*g), imipenem (IPM, 10 *μ*g), amoxicillin/clavulanic acid (AMC 20/10, *µ*g), meropenem (MEM, 10 *μ*g), cefepime (CPM, 30 *μ*g) and aztreonam (ATM, 30 *μ*g). Phenotypic confirmation of ESBL production was performed by double-disc synergy test (DDST). The methodology utilized three discs: AMC, CAZ, and CTX, which were placed 25–30 mm apart with AMC disc in the middle. After overnight incubation at 37°C in air, confirmation of ESBL producing organism was assessed when the zone of inhibition around CAZ and CTX expanded by at least 5 mm close to AMC [7]. ESBL production was also confirmed by Etest (AB Biodisk, Solna, Sweden), using double strips containing CAZ (0.5–32 *μ*g/mL) and CAZ/clavulanic acid (0.064–4 *μ*g/mL), and CTX (0.5–32 *μ*g/mL) and CTX/ clavulanic acid (0.064–4 *μ*g/mL) on Mueller-Hinton agar. Isolates were considered ESBL producers when clavulanic acid resulted in a >3 twofold-concentration decrease (ratio >8) in the MIC. Additionally, a strain was considered an ESBL producer if a phantom zone or a deformed zone around CAZ was observed, independent of the ratios or MICs [7]. ATCC® 35218™ and ATCC® 25922™ *E. coli* control strains were used as positive controls for both beta lactamase and non-beta lactamase producing isolates, consecutively.

### 2.5. Nucleic Acid Extraction and Amplification of Beta Lactamase Genes

DNA was extracted from isolated ESBL producing *E. coli* strains following an overnight growth, using QIAamp DNA Mini Kit (Qiagen GmbH, Hilden, Germany) according to the manufacturer's instructions. All isolates were screened for the resistance genes bla_SHV_, bla_TEM_, bla_CTX-M _by PCR using universal primers (Table 1). PCR amplification reactions were performed in a total volume of 20 *μ*l containing 2 *μ*l of 10 X PCR buffer, 0.5 *μ*l of each forward and reverse primer (10 mM), 1.2 *μ*l of MgCl_2_ (25 mM), 2 *μ*l of dNTP mix (20 mM), 0.5 of AmpliTaq Gold DNA polymerase (Thermo Fisher Scientific, Waltham, Massachusetts) and 2 *μ*l of DNA template. The amplification cycles were as follows: an initial denaturation at 95°C for 15 min; followed by 40 cycles of 95°C for 30 s, 56°C (bla_SHV_, bla_TEM_) or 58°C (bla_CTX-M_) for 30 s, and 72°C for 60 s; and with a final extension at 72°C for 10 min. The amplified PCR products were subjected to electrophoresis at a 1.5% agarose gel in 1 × TAE buffer.

### 2.6. Pulsed-Field Gel Electrophoresis (PFGE)

PFGE was performed using the Xba I restriction enzyme (Thermo Fisher Scientific, Waltham, MA) for *E. coli* isolates (*n* = 35) identified in pregnant women according to the PulseNet protocol. Clonality and genomic relatedness were determined using the CHEF MAPPER (Bio-Rad, Austin, TX). The BioNumeric fingerprinting software (Applied Maths, Belgium) was used to analyze the profile and generate a dendrogram describing the relationship among the isolates. PFGE patterns were analyzed using Tenover's criteria (same cluster if the dice similarity index was >85% and < than 6 bands difference).

### 2.7. Statistical Analysis

Antibiotic susceptibility rates were calculated using frequency and percentages. Colonization with ESBL as independent variable was correlated with the newborn height, weight, and Apgar score (overall assessment of new born well-being used immediately following the delivery of the baby) as dependent variables, taking into consideration other possible confounding variables including mother's age, mother's education, previous miscarriage, delivery week, delivery type, induced labor, recurrent UTI, gestational diabetes, anemia, vaginal discharge, and contact with domestic animals. Beta coefficient, which measures the magnitude of effect of the independent variables on the dependent variable in a multiple regression analysis, was calculated. Statistical significance was calculated using *p*-value and confidence intervals.

The effect of colonization with ESBL on categorical outcome variables (Gestational diabetes, vaginal discharge, induced labor and recurrent UTI) was explored using the test of independence Chi-square. *p*-values were computed considering *p* ≤ 0.05 as significant results.

## 3. Results

### 3.1. Antimicrobial Susceptibility of ESBL Producing Isolates

The present study was conducted on 308 participating pregnant women, 59 (19.1%) ESBL producing Gram-negative bacilli were obtained where the most commonly isolated organism among gram-negative bacilli was *E. coli*, 43 isolates (72.9%), followed by 15 isolates of *K. pneumonia* (25.4%) and one isolate of *Proteus mirabilis* (1.7%).

**Figure 1:**
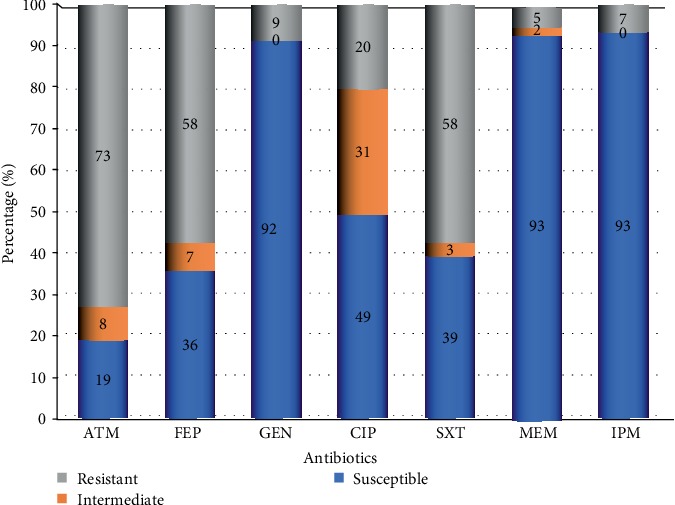
Antimicrobial susceptibility profile of 59 ESBL isolates. Aztreonam (ATM), Cefepime (FEP), Gentamicin (GEN), Ciprofloxacin (CIP), Trimethoprim-sulfamethoxazole (SXT), Meropenem (MEM), Imipenem (IPM).

In this study, all isolates were detected by three phenotypic methods. The result of disc diffusion susceptibility testing of isolated ESBLs revealed that all were resistant to amoxicillin-clavulanic acid, ceftazidime and cefotaxime. The susceptibility rates of isolates to other antibiotics were 39% to co-trimoxazole, 49.2% to ciprofloxacin, 91.5% to gentamicin, 18.6% to aztreonam and 35.6% to cefepime. Most of isolates were highly sensitive to meropenem and imipenem, with a susceptibility of 93.2%. None of isolates was sensitive to all antibiotics and all of isolates showed resistance to more than two antibiotics. The frequency of multidrug-resistant to three and more antibiotics was 25.4% of isolates.  Figure 1 and Table 2 illustrate resistance pattern for different isolated ESBL species among the 59 isolates.

### 3.2. Prevalence of Extended Spectrum Beta Lactamases Genes in E. coli Positive Isolates

PCR was performed on all *E. coli* isolates to detect the most common ESBL *E. coli* producing genes; bla_CTX-M _was the predominant *E. coli* gene (90.7%), followed by bla_TEM_ (88.4%) and finally bla_SHV_ (44.2%) (Table 3). Thirty eight (88.4%) isolates carried more than one type of b-lactamase genes. Coexistence of the bla_CTX-M _and bla_TEM_ was detected in 19 isolates (44.2%), bla_CTX-M _and bla_SHV_ in 3 isolates (6.8%) and bla_CTX-M_, bla_SHV_ and bla_TEM_ in 16 isolates (37.2%). The carriage of a single gene, bla_CTX-M _or bla_TEM_ gene was observed in one and three isolates, respectively.

### 3.3. Pulse Field Gel Electrophoresis Analysis

**Figure 2:**
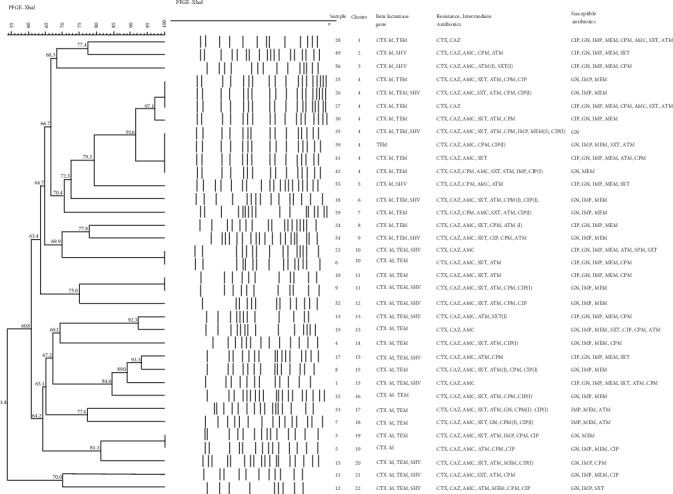
Dendrogram by pulsed-field gel electrophoresis patterns for 34 extended Beta lactamase (ESBL) producing *Escherichia coli*.

PFGE analysis of 34 *E. coli* isolates revealed 22 distinct clusters showing more than 85% similarity. Cluster 4 was prominent in 8 (23%) isolates. Cluster 10, 11, 13, 15, and 19 were seen in more than one *E. coli* isolates. Clutser 4 isolates had different antimicrobial susceptibility profile (Figure 2). Samples 25 and 26 had very similar profiles, the difference between both isolates was the additional presence of SHV gene.

### 3.4. Association between the Presence of Infection and Neonatal Outcomes

In the current study, retrospective data collection showed that two babies born to mothers colonized with ESBL were diagnosed with sepsis. However, both mother and baby isolates were not available for genetic comparison.

Results of the three multiple regression models with neonatal outcomes (weight, height and Apgar score) as dependent variables are displayed in Table 4. Results revealed that ESBL colonization had negative association with height of the newborn, although the association was not statistically significant. However, significant positive association between delivery week and newborn height and significant negative associations between the mother age, delivery type, and newborn height were noted. The height of the newborn increased 0.38 cm with one week increase in delivery time (*p* value = 0.003) and decreased 0.23 cm with the increase in the mothers' age (*p* value = 0.04). Newborn height also decreased with C-section (Beta = 0.46; *p* value = 0.004). On the other hand, ESBL colonization had significant negative association with the weight loss of the newborn (Beta = −0.31; *p* = 0.009). C-section also had significant negative association with weight (Beta = −0.41; *p* value = 0.009), while delivery time had significant positive association with the newborn weight; there was 0.33 g increase in weight with an additional delivery week (*p* value = 0.008). ESBL colonization had negative association with Apgar score, however without statistical significance. The other covariates did not yield significant associations.

## 4. Discussion

The antimicrobial resistance is becoming a major threat in Lebanon. The preexisting colonization of the gastrointestinal track with antibiotic resistant organisms in Lebanese patients has been previously reported [8]. Empirical therapy, inappropriate prescription of antibiotics, and the extensive use of over-the-counter antibiotics aggravated the emergence of antibiotic resistance [9]. In 2013, the prevalence rate of ESBL production of *E. coli* and *Klebsiella *species reached 32.3–29.2%, respectively [10]. In 2016, the percent susceptibility of *Enterobacteriaceae* to third-generation cephalosporins was 59% [11].

This study is the first in Lebanon that evaluates the prevalence of ESBL in pregnant women. Our study showed a 19.1% prevalence, which is similar to previous studies that reported a prevalence that ranges from 7.5% to 25% [12–14]. The high rate of resistant to cefipime, trimethoprim salfamethoxazole, aztreonam, and ciprofloxacin reflects the increasing prevalence of resistance in Lebanon. Gentamycin, meropenem, and impenem were, however, susceptible to most isolated strains and can be used as intrapartum antibiotic prophylaxis for the prevention of the infection. Antibiotic resistant strains may reside in the genital track of the mother and may be transmitted to new born during delivery. Preterm infants are at high risk of ESBL producing *Enterobacteriaceae* sepsis in neonatal care unit. [3, 4, 15]. Maternal-neonatal transmission has been reported, where identical strains were identified from mother to infant through vertical transmission [16–18].

It is not well known whether the usage of antibiotics during the delivery may decrease the risk of acquiring ESBL in new born. There are controversial data showing that antibiotics are able to reach the fetus by crossing the placenta, hence increasing the risk of ESBL-PE acquisition in newborns (ESBL-PE acquisition in neonates). So far, in Lebanon, there is no agreement regarding surveillance of pregnant women for ESBL colonization. screening of pregnant women for species is done only for *streptococcus agalactiae* as a part of antenatal checkup in Lebanon as well as in other middle east countries [19, 20]. Therefore, minimal risk of transmission and infection may reside. The rate of mother-to-infant transmission for other infections should be investigated by conducting further studies that include a continuous collection and screening of mothers' vaginal samples and follow up of their newborn clinical status. In the current study, two babies were diagnosed with neonatal sepsis. In addition, it is also important to conduct long-term follow-up of children born to ESBL infected women who have been exposed to antibiotics before and after delivery. Early diagnosis may therefore minimize the risk of transmission to the baby. It is also essential to study virulence factor such as the K1 capsule that may cause meningitis in neonates.

The risk factor of acquiring ESBL resistance includes the overuse of antibiotics [21], in addition to the transmission of resistance genes from community, livestock, animals, and environment [22]. In the current study, we demonstrated the high prevalence of CTX-M and TEM genes in community patients. The results were consistent with other studies done in the region; however, most of the studies were investigating strains isolated from hospitalized individuals with the common presence of CTX-M-15 gene [23, 24]. CTX-M-producing *E. coli* isolates are often coresistant to various antibiotic classes, which include co-trimoxazole, the aminoglycosides, and the fluoroquinolones [25]. In our study, 18 *E. coli* strains carrying CTX- M gene were resistant to ciprofloxacin and 24 strains were resistant to cotrimoxazole.

PFGE analysis demonstrated a genomic diversity among *E. coli* strains with the presence of one major circulating clone among ESBL producer strains identified in pregnant women. Further analysis should have been done on the neonates in order to investigate if transmission of stain has occurred.

## 5. Conclusion

In conclusion, this study showed that Lebanon has a high prevalence of ESBL carriage in pregnant women. It should be noted that the current study has few limitations; Only pregnant women were examined without conducting a follow-up study on their newborn to measure the rate of transmission. In addition, we did not screen for nonpregnant women which may have enabled the authors to identify if pregnancy could be a risk factor for acquiring resistance strains.

## Figures and Tables

**Table 1:** Primers used for detection of *β*-lactamase genes by PCR.

Gene of resistance	Primer sequence (5′ to 3′)	Fragment size (bp)
bla_CTX-M _	Fwd: AT GTG CAG YAC CAG TAA RGT KAT GGC	593
	RV: TG GGT RAA RTA RGT SAC CAG AAY CAG CGG	

bla_SHV_	Fwd: AGC CGC TTG AGC AAA TTA AAC	713
	RV: ATC CCG CAG ATA AAT CAC CAC	

bla_TEM_	Fwd: C ATT TTC GTG TCG CCC TTA	800
	RV: C GTT CAT CCA TAG TTG CCT GACTTC	

**Table 2:** Percentage of resistance to different antibiotics of three ESBL producing bacteria isolated from pregnant women.

	*E. coli *(*N* = 43)	*K. pneumonia*(*N* = 15)	*P. mirabilis *(*N* = 1)
Aztreonam	*N* = 31 (72%)	*N* = 11 (73.3%)	*N* = 1 (100%)
Cefepime	*N* = 26 (60.5%)	*N* = 7 (46.7%)	*N* = 0 (0%)
Gentamicin	*N* = 4 (9.3%)	*N* = 1 (6.7%)	*N* = 1 (100%)
Ciprofloxacin	*N* = 9 (20.9%)	*N* = 3 (20%)	*N* = 0 (0%)
Trimethoprim-sulfamethoxazole	*N* = 28 (65.1%)	*N* = 5 (33.3%)	*N* = 1 (100%)
Meropenem	*N* = 2 (4.7%)	*N* = 1 (6.7%)	*N* = 0 (0%)
Imipenem	*N* = 2 (4.7%)	*N* = 2 (13.3%)	*N* = 0 (0%)

**Table 3:** Distribution of resistance genes bla_TEM_, bla_SHV_, and bla_CTX-M_ in 43 *E. coli* isolates.

One ESBL gene	bla_CTX-M_	*N* = 1 (2.3%)
	bla_SHV_	*N* = 3 (6.8%)
	bla_TEM_	*N* = 1 (2.3%)

Two ESBL genes	bla_CTX-M _and bla_TEM_	*N* = 19 (44.2%)
	bla_CTX-M _and bla_SHV_	*N* = 3 (6.8%)

Three ESBL genes	bla_CTX-M_, bla_SHV_, and bla_TEM_	*N* = 16 (37.2%)

**Table 4:** Effect of ESBL producing bacteria and other independent confounding variables on height, weight, and Apgar score of the neonate.

	Height			Weight			Apgar score		
	Beta	*p*-value	CI	Beta	*p*-value	CI	Beta	*p*-value	CI
Mother age	−0.23	0.04	−1.79; −0.04	−0.06	0.58	0.58; 165.13	−0.13	0.26	−0.47; 0.12
Previous miscarriage	−0.001	0.99	−0.95; 0.94	−0.07	0.47	0.47; 157.86	0.12	0.27	−0.14; 0.50
Delivery week	0.38	0.003	0.29; 1.31	0.33	0.008	0.008; 316.05	0.03	0.77	−0.15; 0.20
Delivery Type	−0.46	0.004	−3.34; −0.68	−0.41	0.007	0.007; −133.00	−0.21	0.18	−0.75; 0.14
Induced Labor	−0.01	0.88	−1.07; 0.93	−0.04	0.70	0.70; 211.30	−0.06	0.63	−0.42; 0.25
Recurrent UTI	0.09	0.37	−1.27; 3.32	0.16	0.12	0.12; 1058.39	−0.12	0.26	−1.22; 0.33
Gestational diabetes	0.12	0.29	−0.46; 1.50	0.10	0.37	0.37; 369.63	0.17	0.15	−0.09; 0.58
Anemia	0.01	0.89	−1.11; 1.27	0.04	0.69	0.69; 372.19	−0.00	0.99	−0.40; 0.40
Vaginal discharge	−0.004	0.96	−0.87; 0.84	0.12	0.24	0.24; 356.41	−0.16	0.17	−0.49; 0.09
Domestic animals	0.17	0.10	−0.21; 2.38	−0.006	0.95	0.95; 328.79	−0.04	0.72	−0.55; 0.39
Gestational complications	0.18	0.10	−0.17; 1.67	0.25	0.023	0.02; 517.10	0.06	0.59	−0.23; 0.39
ESBL	−0.14	0.22	−1.56; 0.37	−0.31	0.009	0.009; −89.37	−0.14	0.24	−0.53; 0.13

	*R* ^2^ = 0.26			*R* ^2^ = 0.27			*R* ^2^ = 0.18		

Beta: standardized beta coefficient; *R*^2^: coefficient of determination; CI: confidence interval.

## Data Availability

All authors confirm that all data and material are available.
